# THE PREDICTIVE VALIDITY OF SUBJECTIVE SURVIVAL PROBABILITIES AMONG OLDER ADULTS WITH PAIN

**DOI:** 10.1093/geroni/igad104.2589

**Published:** 2023-12-21

**Authors:** Gillian Fennell

**Affiliations:** University of Southern California, Los Angeles, California, United States

## Abstract

Chronic pain, which afflicts 20% of US adults, is associated with heightened mortality risk and lower subjective survival probabilities (SSPs) to advanced ages. However, the accuracy of SSPs for individuals with chronic pain is unknown. To assess whether individuals with and without pain and pain-related activity interference report SSPs that are veridical to their actual lifespans, we exploited multi-wave data from the Health and Retirement Study. Baseline SSPs and covariate data were obtained from the 2000 wave among respondents aged 57-89 with and without pain (N=7,075). SSP reports were then compared to the age at which respondents actually died (if they did) using mortality data from exit interviews in subsequent waves. We conducted a multinomial logistic regression using baseline pain and covariates to predict membership into one of four groups summarizing the accuracy and valence of SSP assessments. The groups were: (1)correctly pessimistic, (2)falsely optimistic, (3)falsely pessimistic, and (4)correctly optimistic. Findings suggest that individuals with baseline pain were significantly more likely to be correctly pessimistic about their longevity with low, but accurate SSPs (falsely optimistic (RRR=.56, p<.001), falsely pessimistic (RRR=.65, p<.001), or correctly optimistic (RRR=.44, p<.001)). This study highlights that chronic pain and associated disability are features of health that individuals use to estimate their own lifespans with greater accuracy. Future research should assess the extent to which this connection is due to self-fulfilling prophecy.

